# Short-Term Effects of Traditional and Alternative Community Interventions to Address Food Insecurity

**DOI:** 10.1371/journal.pone.0150250

**Published:** 2016-03-14

**Authors:** Federico Roncarolo, Sherri Bisset, Louise Potvin

**Affiliations:** 1 Public Health Research Institute, University of Montreal (IRSPUM), Montreal, Quebec, Canada; 2 Public Health School, University of Montreal, Montreal, Quebec, Canada; 3 Canada Research Chair in Community approaches and health inequalities, University of Montreal, Montreal, Quebec, Canada; 4 Agence de la santé et de services sociaux de la Montérégie, Quebec, Canada; 5 Lea Roback Research Centre, Montreal Public Health Directorate, Montreal, Quebec, Canada; University of Reading, UNITED KINGDOM

## Abstract

**Background:**

Despite the effects of food insecurity on health are well documented, clear governmental policies to face food insecurity do not exist in western countries. In Canada, interventions to face food insecurity are developed at the community level and can be categorized into two basic strategies: those providing an immediate response to the need for food, defined “traditional” and those targeting the improvement of participants’ social cohesion, capabilities and management of their own nutrition, defined “alternative”.

**Objective:**

The objective of this study was to evaluate the effects of food insecurity interventions on food security status and perceived health of participants.

**Design:**

This was a longitudinal multilevel study implemented in Montreal, Quebec, Canada. Participants were recruited in a two-stage cluster sampling frame. Clustering units were community organizations working on food insecurity; units of analysis were participants in community food security interventions. A total of 450 participants were interviewed at the beginning and after 9 months of participation in traditional or alternative food security interventions. Food security and perceived health were investigated as dependent variables. Differences overtime were assessed through multilevel regression models.

**Results:**

Participants in traditional interventions lowered their food insecurity at follow-up. Decreases among participants in alternative interventions were not statistically significant. Participants in traditional interventions also improved physical (B coefficient 3.00, CI 95% 0.42–5.59) and mental health (B coefficient 6.25, CI 95% 4.15–8.35).

**Conclusions:**

Our results challenge the widely held view suggesting the ineffectiveness of traditional interventions in the short term. Although effects may be intervention-dependent, food banks decreased food insecurity and, in so doing, positively affected perceived health. Although study findings demonstrate that food banks offer short term reprise from the effects of food insecurity, the question as to whether food banks are the most appropriate solution to food insecurity still needs to be addressed.

## Introduction

Food insecurity, defined as the “limited, inadequate, or insecure access of individuals and households to sufficient, safe, nutritious and personally acceptable food to meet their dietary requirements for a productive and healthy life” [1], represents a major public health concern [2]. The effects of food insecurity on type II diabetes [3, 4] hypertension [4, 5], cardiovascular diseases [4], mental distress [4, 6–9], depression [10] and poor health in general [6, 7, 11] are well documented.

Despite the well-known association between food insecurity and health, food insecurity is steadily increasing in developed countries such as Canada. In 2011–2012, 8.3% of households experienced food insecurity [12] and efforts to stop its growth are thus far unsuccessful [12, 13]. To date, interventions to tackle food insecurity have been developed at the community level and are sometimes institutionalized, but a clear governmental policy to address food insecurity does not exist in Canada nor in the majority of developed countries [14]. This is due to a profound shift in federal and provincial social policy from a welfare state with modest social rights established in the period 1966–73 to market-driven, neo-liberal approaches[15]. This shift first began in Canada and in many western countries in 1981 and intensified during the following two decades. Indeed, the influence of neoliberalism has marked a return to privatization and charitable or faith-based responses to basic human needs such as food and shelter [15]. Notwithstanding these efforts from community-based organizations they are described as insufficient and inadequate [16], although scientific evaluation of the effectiveness of such interventions has never been published. This research aimed to provide knowledge concerning the effectiveness of community-based food insecurity interventions.

Interventions in food insecurity implemented by community organizations can be categorized into two basic strategies: those facilitating access to food by providing an immediate response to the need for food and those targeting the improvement of participants’ social cohesion, capabilities and management of their own nutrition. The traditional type of intervention, represented by food banks, is oriented toward the satisfaction of immediate needs of people experiencing food insecurity through food supply. Food banks targeting routine food necessities are the most immediate response to food insecurity and are ideally short term solutions [17]. The second intervention strategy, providing an alternative to the traditional, are represented by community kitchens, community gardens and buying groups. These interventions have objectives related to empowerment, supporting the development of skills that allow participants to steadily improve their food insecurity status. The objective of alternative interventions reaches beyond food insecurity problem and involves aspects of social inclusion, social capital and participation in civic activities. Neither traditional nor alternative food security interventions pursue the objective of impacting the economic factors at the root of food insecurity, nor the broader systemic factors that shape food production and distribution. It has therefore been suggested that these interventions have limited potential to impact food insecurity status[18].

The role of community interventions in facing food insecurity is at the core of a long-standing debate. Some researchers argue that food banks exacerbate rather than alleviate food insecurity by masking it, undermining social justice and relieving governments of their duties [16, 19, 20]. In contrast, other researchers underline the importance of food banks, affirming the importance of their role in addressing hunger and health issues[21, 22]. In this respect, it is argued that the strategic position community organizations have in changing food insecurity intervention strategies may be strengthened.

Despite this important debate regarding how public policy and intervention may or may not eradicate food insecurity, there is a lack of empirical evidence about the effectiveness of the different intervention strategies commonly implemented by community organizations to address food insecurity. Indeed, in spite of the multitude of studies correlating food insecurity and health, there is a lack of data on the effectiveness of interventions on households’ food insecurity and their effects on participants’ health [23]. The rare studies analyzing the health of participants in food insecurity interventions were based on interviews during or at the end of the interventions, generally through retrospective accounts often based on a small number of respondents [24, 25]. The relationship between food insecurity and health is complex and recursive: food insecurity can cause ill-health and poor health can worsen food insecurity [26]. In addition, the mediating effect of participation in food insecurity interventions has not been addressed.

To our knowledge, this is the first study that selected and recruited new participants from community-based food insecurity interventions for a prospective effectiveness evaluation. The objective of this study is to evaluate the short-time effects of food insecurity interventions on food security and perceived health of participants. Identifying the effects of interventions on health and food insecurity is essential to advance knowledge regarding the effectiveness of food insecurity intervention overall, as well as with respect to traditional and alternative approaches.

## Methods

### Sample Selection

This is a longitudinal study of newly recruited participants in traditional and alternative interventions. Participants were recruited in a two-stage cluster sampling frame. Clustering units were community organizations working on food insecurity in the Montreal Metropolitan Region (MMR). The MMR includes 82 municipalities and a population of nearly four millions people [27]. The list of organizations involved in food insecurity interventions was validated through the confirmation of experts with in-depth knowledge of the food insecurity intervention network in the Montreal area. Organizations exclusively targeting children such as school lunch and breakfast programs were excluded. A total of 451 organizations were selected. We administered a phone survey to the directors of 30.1% (136 / 451) of these organizations, randomly selected, to identify the interventions each organization implemented and the number of new and overall participants. Of the 136 surveyed organizations, 61 uniquely offered traditional interventions and 75 offered a form of alternative intervention. Organizations offering both traditional and alternative interventions were classified as alternative. Participants selected from these organizations were uniquely participating in alternative interventions

Organizations were invited to participate in our study based upon their number of new participants each year. A minimum number of 30 and 50 participants having begun a food insecurity intervention in the past 6 months was set respectively for alternative and traditional interventions. This criteria for participation was determined first, by considering what could be expected in terms of participant recruitment and second, what was needed in terms of statistical analysis. Community partners’ expertise first informed our criteria for the minimum number of new participants. Namely, new participants in alternative interventions are less numerous than in traditional interventions whereas participation is stable and constant for longer periods of times. While the number of new participants in traditional interventions is higher, participation is more sporadic and related to temporary situations. Second, power calculations for the minimum number of participants required for statistical analysis, as further explained below, considered the hierarchical nature of the data.

Individuals between 18 and 65 years of age registered for the first time, and for less than 6 months in selected MMR food insecurity community organizations were invited to participate in our study. People older than 65 years of age were excluded from the study because, in Québec, they can benefit from income supplement and have preferential paths to fight food insecurity. Homeless people were also excluded for two reasons. First, homeless people represent a sub-population extremely vulnerable and their strategies to cope with food are different from the rest of the population [28]. Second, their inclusion in the study would have biased results because of the lack of long term strategies for food insecurity directed to this vulnerable population.

### Multi-level modeling

Services and resources provided by organizations, although classified in the same category (i.e. food bank) could differ among organizations according to policies, quality of food or frequency of access. Participants in our study were nested within organizations. With nested data observations may not be independent and hierarchical multilevel modeling is recommended [29].

To account for these differences along with the longitudinal nature of the data, analysis accounted for data structured into three levels. The first level was the change overtime in dependent variables, the second level were individuals, the third corresponded to the organization where participants were recruited. With this hierarchical structure of data, and since the intraclass (among individuals) and interclass (among different organizations) coefficients for our outcome variables were unknown, we needed a large number of participants in each organization to detect a 10% difference in the variables measuring food security with an acceptable degree of precision (0.9) and at a statistically significant threshold (0.05). Among the 136 organizations, 16 organizations carrying out traditional interventions and 6 implementing alternative interventions met the criteria for a minimum number of new registered participants.

### Measures and Variables

From October 2011 to May 2012 a questionnaire to investigate health, food insecurity and vulnerability was completed by participants with the support of research assistants specifically-trained to accompany participants through the completion of the questionnaire. The questionnaire took approximately 30–45 minutes and was administered face to face in French or in English, according to the preference of participants. Interviews took place in the organizations providing food insecurity interventions or in the nearby area. Participants were informed that they would be called back and invited to participate in the second part of the study and were asked to inform the research team in case of changes of address. Six months after the first interview, a postcard inviting participants to communicate possible changes of address was sent to each participant. Nine months after the first interview, participants were invited by phone to complete the follow up interview. A nine month follow-up was considered adequate to detect intervention effects on the level of food security and health status because according to community partners, sufficient time has passed such that participation in alternative food security interventions has become regular. Non respondents were contacted by mail and invited to contact project managers. The second interview took place in the same location as the first. In case of changes of address or impracticability of the first location, another place was chosen by mutual agreement. The same questionnaire was used for the first and the second survey.

The categorizing variable corresponded to participants’ enrollment in one of the two intervention strategies, traditional or alternative. Dependent variables were food security status and perceived physical and mental health. Control variables considered in the study were gender, age, country of birth, marital status and income.

#### Food security status

Food security status was measured using the food security module included in the Canadian Community Health Survey(CCHS)[30]. The food security module presents the same questions used in the United States Household Food Security Survey Module (HFSSM). The HFSSM was validated to measure change in food security status overtime [31]. The CCHS calculates three scores of food insecurity for the previous year: for the respondent, for dependent children (when applicable) and for the respondent’s household. The CCHS food security module is composed of 10 adult-related and 8 children-related questions investigating whether the respondent or other household members experienced indicators of food insecurity. Questions query the severity of the experiences associated with food, such as an anxiety that food will run out, a need to modify the amount of food consumed, experiencing hunger, and in the extreme, going a whole day without eating.

Each multiple choice answer is recoded scoring 0 or 1 point, where 0 corresponds to food security and 1 to food insecurity status. For example, if the question “You and other household members worried that food would run out before you got money to buy more. Was that often true, sometimes true, or never true in the past 12 months?” was answered “never true” the question was coded as 0 while responses of “often true” or “sometimes true” were coded 1. The answers “often” and “sometimes” are both considered affirmative responses because they indicate that the condition occurred at some time during the year [31].

The final score ranges between 0 to 10 for adults and 0 to 8 for children. The food security module defines three levels of food security: food security, with scores of 0 or 1, moderate insecurity with a score between 2 and 5 for adults and 2 and 4 for children, and severe insecurity with score respectively above 5 for adults and above 4 for children. Household food security status is dependent on both adult and child scores. In families with children, the household is food secure if both adults and children are food secure; the household is moderately food insecure if either adults or children are moderately food insecure but neither is severely food insecure; the household is severely food insecure if either adults or children are severely food insecure. In childless households, adults’ food security status corresponds to household food security status.

#### Health related quality of life

Generic health-related quality of life was measured using the SF-12-v2 questionnaire [32]. The SF-12-v2 is a shorter and validated version of SF-36, regularly used to assess perceived health [33]. The questionnaire tests physical and mental health in the last four weeks generating 8 subscales: 1- physical functioning (composed of 2 items: health limitations in accomplishing moderate activities such as moving a table, or pushing a vacuum cleaner; health limitations in climbing several flights of stairs); 2- role limitations due to physical problems (composed of 2 items: limitations accomplishing what one desires to accomplish; limitations in the kind of work or activities); 3- bodily pain (composed of 1 item: pain interference in the accomplishment of normal work); 4- general health perceptions (composed of 1 item: own health perception); 5- vitality (composed of 1 item: perceived energy); 6- social functioning (composed of 1 item: interference of physical health or emotional problems); 7- role limitations due to emotional problems (composed of 2 items: limitations in accomplishing what wanted as a result of feeling depressed or anxious; less attention in doing work or other activities); and 8- mental health (composed of 2 items: perception of calm and peace; perception of downhearted and depression). Based on these subscales, two summary scores were calculated: the physical (PCS) and the mental (MCS) component summary scores. PCS was built with the subscales 1 through 4 and MCS was built with subscales 5 through 8.PCS and MCS scores were transformed in a 0–100 score according to published regression weights and scoring rules (a higher score indicating better health-related quality of life) as suggested in the user manual.[34]

#### Household income and other control variables

Respondent’s gender, age, country of birth, being part of a visible minority, marital status and household income were self-reported. According to the Employment Equity Act of Canada, we defined visible minorities "persons, other than Aboriginal peoples, who are non-Caucasian in race or non-white in colour". Household income was grouped into 7 categories ranging from “no income” to “income superior to $40000”.

### Statistical analysis

In our analysis, missing values were not imputed but excluded pair-wise. Since our data were repeated measures from individuals nested in organizations and categorized in two different interventions, the influence of dependent variables on the outcomes (food insecurity and health) was investigated with multilevel regression analyses.

We used multilevel models to account for the hierarchical structure of data and likewise, to avoid an underestimation of the group effect and incorrectly rejecting the null hypothesis of no difference (i.e. type I error)[29]. The analyses were executed separately for participants in traditional and alternative interventions. Generalized linear latent regression models (GLLAMM) were used. GLLAMM performs maximum likelihood estimation by using adaptive quadrature. Three-level random intercept regression models were constructed for food security for each intervention strategy. A first model was constructed using food insecurity as the dependent variable. Subsequently, a sequence of controlling variables (respondent’s gender, age, country of birth, marital status and income) were entered as covariates at the individual level of the model. No organization level factors were added to the models.

Six linear random intercept regression GLLAMM models were used to assess perceived health (three for physical health and three for mental health) in each intervention group. The first two models considered physical and mental health unadjusted, the following two models were adjusted for respondents’ gender, age, country of birth, marital status and income, while the last two models were also adjusted for adults’ food security status. Differences across time in the two intervention groups were also tested through GLAMM models. The STATA v11.2 software was used to perform statistical analysis.

### Ethic statement

This study was conducted according to the guidelines laid down in the Declaration of Helsinki and all procedures involving human subjects/patients were approved by the health research ethics committee (CERES) of the University of Montreal. Written informed consent was obtained from all subjects/patients.

## Results

In total, 824 new participants responded to the first questionnaire; 711 were participants in one of the 16 organizations classified as offering uniquely traditional interventions, and 113 were participants in one of the 6 organizations offering alternative interventions.

**Fig 1:**
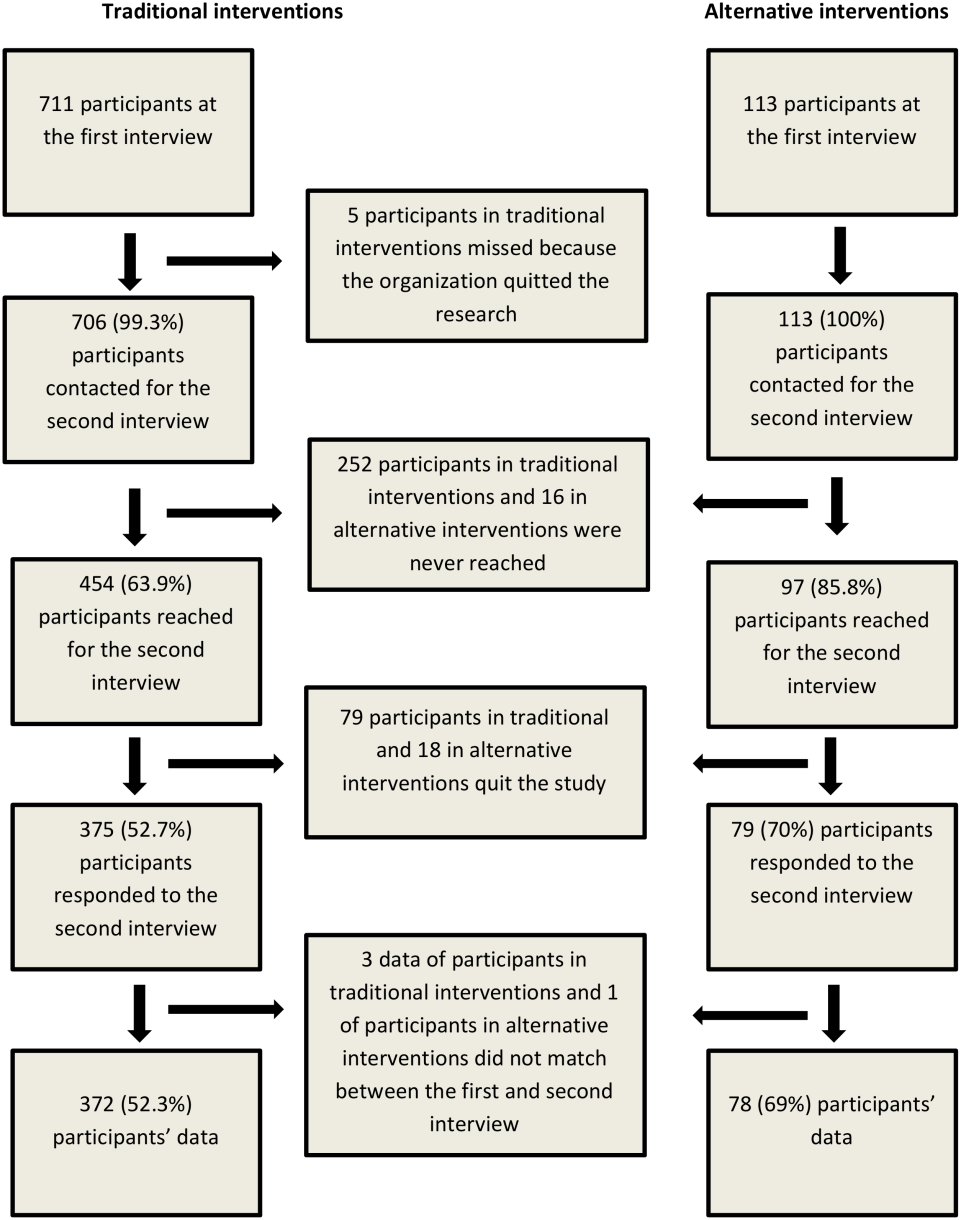
Data on participation and missing at follow-up for traditional and alternative interventions.

Among the 824 original respondents, 374 (45.4%) participants were missing at follow up (Fig 1).

The final sample size was composed of 450 people: 372 participants in traditional interventions (52.3% of initial participants) and 78 participants in alternative interventions (69% of initial participants). No statistical significant differences were found in the descriptive characteristics of participants according to loss to follow up (Table 1; Chi square tests were performed to assess differences). Statistically significant differences between the two intervention groups were found with respect to gender (p = 0.000), country of birth (p = 0.000), belonging to a visible minority (0.014) and marital status(p = 0.001). More in depth analysis of the differences between the two study populations have been described elsewhere. [35, 36]. Intra-group statistically significant differences were found in employment status and income when we compared the first and the second interview data: participants were more likely to be employed and to have a higher income at follow-up. These differences in employment status were evident in both groups (traditional interventions, p = 0.000; alternative interventions p = 0.010), while differences in income were evident only within traditional intervention participants (p = 0.001) (Table 2).

**Table 1:** Descriptive characteristics of new participants in traditional and alternative interventions.

	Participants in traditional interventions n. 372 (%)	Participants in alternative interventions n. 78 (%)	Total n. 450 (%)
Gender*			
Male	171 (46.0)	19 (24.4)	190 (42.2)
Female	201 (54.0)	59 (75.6)	260 (57.8)
**Age**			
<30 years	43 (11.6)	13 (16.9)	56 (12.5)
30–49 years	190 (51.2)	40 (51.9)	230 (51.3)
50–65 years	138 (37.2)	24 (31.2)	162 (36.2)
Country of birth*			
Canada	252 (67.3)	36 (46.2)	288 (64.1)
Others	119 (32.1)	42 (53.8)	161 (35.9)
Visible minority*			
Yes	85 (23.7)	29 (37.2)	114 (26.1)
No	274 (76.3)	49 (62.8)	323 (73.9)
Marital status*			
Married/ common law spouse	118 (32.2)	30 (39.5)	148 (33.4)
Single	174 (47.4)	22 (28.9)	196 (44.2)
Other (separated, divorced, widowed)	75 (20.4)	24 (31.6)	99 (22.3)

Age and marital status refer to the first interview

* p value <0.05

**Table 2:** Descriptive characteristics of new participants in traditional and alternative interventions.

	Participants in traditional interventions n. 372 (%)		Participants in alternative interventions n. 78 (%)	
	T1	T2	T1	T2
**Employment status**				
Working	29 (7.8)	73 (19.6)	9 (11.5)	19 (24.4)
Studying	33 (8.9)	29 (7.8)	9 (11.5)	11 (14.1)
Working and studying	11 (3.0)	16 (4.3)	3 (3.8)	3 (3.8)
At home	245 (65.9)	182 (48.9)	51 (65.4)	34 (43.6)
Other	8 (2.2)	69 (18.5)	2 (2.6)	11 (14.1)
Not respondent	46 (12.4)	3 (0.8)	4 (5.1)	0
**Education**				
Less than a high school diploma	98 (26.3)	102 (27.4)	19 (24.4)	12 (15.4)
Secondary (high) school diploma or equivalent	94 (25.3)	97 (26.1)	18 (23.1)	17 (21.8)
Less than a bachelor degree	105 (28.2)	99 (26.6)	19 (24.4)	23 (29.5)
Bachelor’s degree or above	62 (16.7)	71 (19.1)	21 (27.0)	26 (33.3)
Not respondent	13 (3.5)	3 (0.8)	1 (1.3)	0
**Income**				
<5000$	39 (10.5)	16 (4.3)	8 (10.3)	6 (7.7)
5000–9.999$	119 (32.0)	118 (31.7)	11 (14.1)	13 (16.7)
10000–14.999$	102 (27.4)	111 (29.8)	17 (21.8)	21 (27.0)
15000–19.999$	26 (7.0)	42 (11.3)	9 (11.5)	8 (10.3)
20000–29.999$	19 (5.1)	41 (11.0)	10 (12.8)	11 (14.1)
30000–39.999$	12 (3.2)	16 (4.3)	6 (7.7)	5 (6.4)
≥40000$	14 (10.8)	10 (2.7)	7 (9.0)	6 (7.7)
Not respondent	41 (11.0)	18 (4.8)	10 (12.8)	8 (10.3)

Participants in traditional interventions had lowered their food insecurity at follow-up. This was true for households with our without children. Decreases among participants in alternative interventions were not statistically significant (Table 3).

**Table 3:** Food security status traditional and alternative interventions participants distinguishing among adults, children and household.

**A**				
**Participants in traditional interventions n.372**	**T1**	**T2**	**OR (CI)**	**Adjusted OR (CI)** ^a^
**Adults**				
Food secure	41 (11.6)	86 (23.4)	Ref	Ref
Moderate insecure	138 (39.0)	141 (38.3)	0.23 (0.12;0.46)	0.30 (0.14;0.62)
Severe insecure	175 (49.4)	141 (38.3)	0.18 (0.09–0.36)	0.22 (0.10;0.44)
**Children**				
Food secure	33 (24.3)	58 (40.3)	Ref	Ref
Moderate insecure	79 (58.1)	74 (51.4)	0.37 (0.18;0.76)	0.39 (0.17;0.89)
Severe insecure	24 (17.6)	12 (8.3)	0.20 (0.08; 0.51)	0.22 (0.08;0.64)
**Household**				
Food secure	37 (10.4)	77 (20.9)	Ref	Ref
Moderate insecure	141 (39.8)	148 (40.2)	0.29 (0.15;0.55)	0.39 (0.20;0.77)
Severe insecure	176 (49.7)	143(38.9)	0.22 (0.12;0.42)	0.27 (0.14;0.54)
**B**				
**Participants in alternative interventions n.78**	**T1**	**T2**	**OR (CI)**	**Adjusted OR (CI)** ^a^
**Adults**				
Food secure	23 (32.4)	30 (40)	Ref	Ref
Moderate insecure	28 (39.4)	28 (37.3)	0.60 (0.23;1.54)	0.36 (0.10;1.31)
Severe insecure	20 (28.2)	17 (22.7)	0.51 (0.18; 1.41)	0.32 (0.08;1.25)
**Children**				
Food secure	16 (45.7)	21 (60.0)	Ref	Ref
Moderate insecure	15 (42.9)	12 (34.2)	0.33 (0.07;1.62)	0.35 (0.07;1.70)
Severe insecure	4 (11.4)	2 (5.7)	0.20 (0.02; 1.85)	0.21 (0.01; 8.33)
**Household**				
Food secure	23 (32.4)	29 (38.7)	Ref	Ref
Moderate insecure	26 (36.6)	29 (38.7)	0.70 (0.26;1.83)	0.42 (0.11;1.53)
Severe insecure	22 (31.0)	17 (22.7)	0.48 (0.17;1.35)	0.32 (0.08;1.25)

^a^ OR is adjusted for respondent’s gender, age, country of birth, marital status and income.

Participants in traditional interventions reported improved physical (adjusted B coefficient 3.00, CI 95% 0.42–5.59) and mental health (adjusted B coefficient 6.25, CI 95% 4.15–8.35) at follow-up. However, improvement in physical health disappeared when adjusting for food security. No change in physical health at follow-up was found for participants in alternative interventions. Mental health improvement was found among participants in alternative interventions, however this effect disappeared in the adjusted model, which is likely due to the study’s lack of power (Table 4). No statistical significant differences were found between participants in traditional and in alternative interventions across time.

**Table 4:** Perception of physical and mental health traditional and alternative interventions participants. Scores are in percentage scale.

**A**					
**Participants in traditional interventions n. 372**	**T1**	**T2**	**β coefficient (CI)**	**Partially adjusted β coefficient (CI)** ^a^	**Adjusted β coefficient (CI)** ^b^
**Physical Components Score**	63.97	66.91	3.02 (0.63;5.40)	3.00 (0.42;5.59)	1.51 (-1.11;4.12)
**Mental Components Score**	58.13	63.86	5.85 (3.92; 7.78)	6.25 (4.15; 8.35)	5.28 (3.13; 7.42)
**B**					
**Participants in alternative interventions n. 78**	**T1**	**T2**	**β coefficient (CI)**	**Partially adjusted β coefficient (CI)** ^a^	**Adjusted β coefficient (CI)** ^b^
**Physical Components Score**	70.62	70.59	0.11 (-4.93; 5.15)	-1.10 (-6.57; 4.36)	-0.89 (-6.66;4.89)
**Mental Components Score**	66.06	71.1	4.66 (0.10; 9.23)	4.51 (-0.39; 9.41)	4.21 (-1.28;9.69)

^a^ β coefficient is adjusted for respondent’s gender, age, country of birth, marital status and income,

^b^ β coefficient is adjusted for respondent’s gender, age, country of birth, marital status, income, and food security status

## Discussion

The objective of this study was to evaluate the short term effects of two distinct food insecurity intervention approaches, traditional and alternative, on participants’ food security and perceived health. Study results found traditional, but not alternative food insecurity interventions to have short term effects on participants. Nevertheless, although not statistically significant, participants in alternative interventions also improved their food insecurity. Relative to their baseline measures, participants in traditional interventions reported a decrease in food insecurity and improvement in self-reported mental and physical health at nine-month follow-up. No such improvements were reported for participants in alternative interventions and while not statistically significant, an improvement was reported for mental health.

The absence of statistically significant effects on food insecurity and perceived health among alternative interventions is not surprising for several reasons. First, people who start participating in alternative interventions are generally more food secure and less vulnerable than those who start participating in traditional interventions. [35, 36] Moreover, collective kitchens and community gardens do not have short term objectives with regard to food insecurity and health. Rather, alternative interventions aim to decrease food insecurity through mutual collaboration, empowerment and social inclusion; they implement long term strategies building change over time [18]. Therefore, although they are often identified as food insecurity interventions, their potential effects go beyond food insecurity alone. Moreover, in our study, the sample of participants in alternative interventions may have been too small to allow enough power to detect significant effects. A small number of participants in alternative food insecurity interventions is associated with the hierarchical nature of our data and the need to employ strict inclusion criteria. Receiving 30 new participants in the 6 months that preceded baseline measurements did not occur for most organizations that offered alternative interventions and many could not guarantee the minimum number of participants.

The short-term impact of food banks on perceived health confirmed the already known association between food insecurity and health. [5–9, 11, 37] Nevertheless, this research adds important facets to this association.[35, 36] First, results show that 9 months of intervention are sufficient to show decreases in food insecurity and improvement in perceived mental and physical health. It appears thus, that improvements in food insecurity are concomitant with improvements in health. This finding underlines the importance of acting swiftly in facing food insecurity. Many households do not have access to food banks because of lack of information, perceptions of food aid, or feelings they are not in enough need [16]; a prompt participation in food banks may rapidly improve food insecurity and consequently their health. Secondly, the use of traditional interventions was found to have a positive association with mental health even after adjusting for food insecurity. Over and above any indirect effect from improving food insecurity status, participating in a traditional type of food insecurity intervention appears to have a direct and positive effect on mental health. The use of food banks may guarantee the family’s regular access to food and this may impact feelings of alienation characterizing people in food insecurity [38] and consequently improve mental health. Nevertheless, participants often have to overcome feelings of shame, embarrassment, degradation and humiliation before accessing food banks [39–41]. This finding of a positive association between the use of traditional intervention and mental health improvements provides an additional argument for why food banks need to be made easily accessible to any household in food insecurity and the importance of overcoming the resistance and inability that people in food insecurity have in accessing food banks [40, 41].

Some study limitations should be taken into account in the discussion of this study’s results. At first, we limited our selection criteria to organizations with at least 50 newly registered subjects within 6 months for traditional strategy, 30 for alternative strategy. This criterion excluded the smallest organizations. Participants in small organizations could present different characteristics relative to those participating in bigger organizations. Moreover, this criterion limited the number of organizations implementing alternative interventions that could be enrolled in the study. This limited the statistical power in data analysis, especially concerning alternative interventions. A second limitation is related due to the high rate of loss to follow-up; nevertheless, our retention rates are in line with other studies on vulnerable populations[42, 43].

Our results challenge the widely held view suggesting the ineffectiveness of traditional interventions, at least in the short term. Given the nature of traditional food insecurity interventions, effects are likely to disappear when participants are no longer exposed to the intervention. This is however congruent with the objectives of food banks, which is to provide first aid intervention and decrease participants’ food insecurity over a limited period of time.

## Conclusions

Traditional interventions represent an effective food security intervention in short-term, improving both food insecurity status and health of participants. Nevertheless, these positive effects may be time-limited and disappear when access to traditional interventions ends. Although food banks may offer a short term reprise from the effects of food insecurity, the question as to whether food banks are an appropriate solution, socially and politically, to food insecurity should not be overlooked. As clearly stated in recent food banks reports, it is a mistake to think that food banks or other charitable food programs are able to adequately address household food insecurity over the long term [41, 44]. Further research is needed to identify how traditional interventions have an effect upon long term food insecurity and the potential that alternative interventions may offer longer term advantages.
